# Efficacy of chemotherapy in metastatic male breast cancer patients: a retrospective study

**DOI:** 10.1186/s13046-015-0143-8

**Published:** 2015-03-21

**Authors:** Luigi Di Lauro, Laura Pizzuti, Maddalena Barba, Domenico Sergi, Isabella Sperduti, Marcella Mottolese, Pietro Del Medico, Franca Belli, Patrizia Vici, Ruggero De Maria, Marcello Maugeri-Saccà

**Affiliations:** Division of Medical Oncology B, “Regina Elena” National Cancer Institute, Via Elio Chianesi 53, 00144 Rome, Italy; Scientific Direction, “Regina Elena” National Cancer Institute, Via Elio Chianesi 53, 00144 Rome, Italy; Biostatistics Unit, “Regina Elena” National Cancer Institute, Via Elio Chianesi 53, 00144 Rome, Italy; Department of Pathology, “Regina Elena” National Cancer Institute, Via Elio Chianesi 53, 00144 Rome, Italy; Division of Medical Oncology, Reggio Calabria General Hospital, Reggio Calabria, Italy; Division of Oncology, Spolverini Hospital, Ariccia, Italy

**Keywords:** Male breast cancer, Metastatic disease, Chemotherapy, Anthracycline-containing regimens, Anthracycline-free regimens

## Abstract

**Background:**

The role of chemotherapy in the treatment of metastatic male breast cancer patients remains unknown, and the only available evidence stem from small, retrospective series evaluating outdated drugs and/or regimens.

**Methods:**

In this retrospective study we evaluated the activity of polychemotherapy, consisting of three-drug (anthracycline-containing and anthracycline-free) regimens, as a first-line therapy for metastatic male breast cancer patients who had received at least one prior endocrine therapy.

**Results:**

Fifty patients treated between 1978 and 2013 were included in the present analysis. Regarding best response, we recorded 1 (2%) complete response and 27 (54%) partial responses, for an overall response rate of 56% (95% CI, 42.2-69.8). Considering stable disease, the disease control rate was 84%. Median progression-free survival was 7.2 months (95% CI, 5.9-8.5), and median overall survival was 14.2 months (95% CI, 12.2-16.2). Albeit we observed some differences for all the outcomes explored when comparing anthracycline-containing and anthracycline-free regimens, they were not statistically significant.

**Conclusions:**

Chemotherapy, consisting in both anthracycline-containing and anthracycline-free regimens, showed encouraging antitumor activity in metastatic male breast cancer patients previously treated with endocrine therapy.

## Background

Male breast cancer (MBC) is a rare disease accounting for less than 1% of all breast cancer (BC) cases [1]. Patients who develop a metastatic disease are mainly treated with anti-hormone therapies [2]. Initial hints on the therapeutic potential of manipulating the hormonal background dates back to the 1940s when endocrine surgery (orchiectomy, adrenalectomy and hypophysectomy) was associated with tumor regressions [3]. The use of hormonal treatments has found more concrete ground with investigations aimed at providing molecular information to assist in clinical decision-making [4]. Collectively, estrogen and progesterone receptors were detected as often expressed in MBC, even more frequently than in female BC (FBC) [4]. A therapeutic role for the androgen receptor was more recently envisioned based on immunohistochemical and gene expression profile studies [5,6].

Thus, the current treatment paradigm for metastatic MBC patients (mMBC) relies on the concept of delaying chemotherapy as long as possible with the use of sequential anti-hormonal treatments. A number of factors account for this approach. Firstly, the wealth of hormonal medical treatments available, including tamoxifen [7], aromatase inhibitors [8-11], fulvestrant [12,13] and anti-androgens [14-16]. Though retrospectively, all the afore-mentioned compounds showed clinical activity [7-16]. Secondly, the lesson we learned from FBC is that chemotherapy is overall less effective in endocrine-responsive tumours. Thirdly, MBC is a disease of elderly men [1], for whom the harm possibly deriving from chemotherapeutic agents along with the often co-existing comorbidities refrain from using chemotherapy. Finally, information gathered on the use of chemotherapy in this population are scarce and stem from retrospective, small-sized studies either reporting on outdated drugs/regimens [17,18] or with no clear focus on chemotherapy efficacy [19].

Nevertheless, the natural history of the disease, during which adaptive changes arisen following prolonged drug administration render cancer cells no longer dependent on hormonal stimuli, forces clinicians to consider chemotherapy. In this context, however, uncertainty dominates, and daily clinical management of patients no longer benefiting from anti-hormone treatments is largely empirical. Owing to the gap existing in current medical literature, we herein describe our clinical experience with polychemotherapy in the treatment of mMBC patients.

## Methods

The study population was composed by 50 metastatic MBC patients who received polychemotherapy. All patients had received at least one prior hormonal treatment in the metastatic setting. All patients were treated between 1978–2013. The majority of patients was clinically managed at the “Regina Elena” National Cancer Institute, Rome. Medical records were reviewed in order to obtain information on demography, molecular pathology, treatment administered and outcomes. Patients received the following regimens: 5-fluorouracil, doxorubicin and cyclophosphamide (FAC) 500/50/500 mg/m^2^ every 3 weeks, 5-fluorouracil, epirubicin and cyclophosphamide (FEC) 500/75/500 mg/m^2^ every 3 weeks, docetaxel, epirubicin and cyclophosphamide (TEC) 75/75/500 mg/m^2^ every 3 weeks, intravenous cyclophosphamide, methotrexate and 5-fluorouracil (CMF/iv) 600/40/600 mg/m^2^ on days l and 8 every 4 weeks, or oral cyclophosphamide, methotrexate and 5-fluorouracil (CMF/oral) 100/40/600 with cyclophosphamide given orally d1-14. Treatment was continued until disease progression, unacceptable toxicity, death, and for a maximum of 6 cycles. As our patients were treated over a period of ~35 years, tumor response was evaluated according to the criteria outlined by the International Union Against Cancer, the World Health Organization [20,21] or the Response Evaluation Criteria In Solid Tumors (RECIST 1.1). Progression-free survival (PFS) and overall survival (OS) were calculated from the date of therapy initiation to the date of disease progression or death from any cause, respectively. PFS and OS were analyzed according to the Kaplan-Meier method. Comparisons between regimens were performed using the log-rank test. All statistical analyses were performed using SPSS statistical software version 20 (SPSS inc., Chicago IL, USA). This retrospective study was approved by the Ethic Committee of “Regina Elena” National Cancer Institute of Rome, the coordinating centre, and was carried out according to the Helsinki Declaration.

## Results

Fifty patients (median age: 66 years, range: 24–78) treated between 1978 and 2013 were included in this study. Patients’ characteristics are illustrated in Table 1. All patients had received at least one prior hormonal treatment for their metastatic disease. In the first-line setting 24 patients received cyproterone acetate (either as a monotherapy or combined with a GnRH analogue), 15 patients received letrozole with a GnRH analogue, 2 letrozole, 2 anastrozole, 6 tamoxifen and 1 exemestane. The median number of prior therapy with anti-hormonal agents for advanced disease was 1 (range 1–3). Forty-eight tumors (96%) were estrogen and/or progesterone receptor-positive. HER2 status was negative or unknown in all tumors. Thirty-eight patients (76%) had visceral metastases. None of them had brain metastases at the beginning of chemotherapy. Forty patients (80%) had 2 or more metastatic sites.

**Table 1:** **Baseline characteristics in metastatic male breast cancer patients treated with first-line chemotherapy following endocrine therapy (N = 50)**

**Characteristic**	**N**	**%**
Age		
Median	66	-
Range	24-78	-
ECOG PS		
Median	1	-
Range	0-2	-
Hormone receptor status		
Positive	48	96
Unknown	2	4
HER2 status		
Negative	19	38
Unknown	31	62
Adjuvant CT		
Yes	5	10
No	45	90
Lines of HT for advanced disease		
Median	1	-
Range	1-3	-
Dominant disease site		
Visceral	38	76
Bone	10	20
Soft-tissue	2	4
Number of disease sites		
1	10	20
2	25	50
≥3	15	30
Chemotherapy regimens		
FAC	21	42
FEC	11	22
TEC	3	6
CMF (*intravenous*)	10	20
CMF (*oral*)	5	10

ECOG PS: Eastern Cooperative Oncology Group Performance Status; CT: chemotherapy; HT: hormonal therapy; FAC: Fluorouracil, Doxorubicin and Cyclophosphamide; FEC: Fluorouracil, Epirubicin and Cyclophosphamide; TEC: Docetaxel, Epirubicin and Cyclophosphamide; CMF: Cyclophosphamide, Methotrexate and Fluorouracil.

Overall response rate (ORR) was 56% (95% CI, 42.2-69.8). In detail, we recorded 1 (2%) complete response (CR) in a patient with liver and skin metastases treated with TEC, and 27 (54%) partial responses (PR). Stable disease (SD) was observed in 14 patients (28%). Disease control rate (DCR), defined as CR + PR + SD, was 84%. Progressive disease (PD) was seen in 8 patients (16%). ORR was 60% in patients treated with anthracycline-containing regimens and 46.7% in patients treated with anthracycline-free regimens (Table 2).

**Table 2:** **Objective response to first-line chemotherapy in metastatic male breast cancer (N = 50)**

**Responses**	**Overall**		**Antra-based chemotherapy**		**Non antra-based chemotherapy**	
	**N**	**%**	**N**	**%**	**N**	**%**
**Complete response**	1	2	1	2.9	-	-
**Partial response**	27	54	20	57.1	7	46.7
**Stable disease**	14	28	9	25.7	5	33.3
**Progressive disease**	8	16	5	14.3	3	20

**Figure 1:**
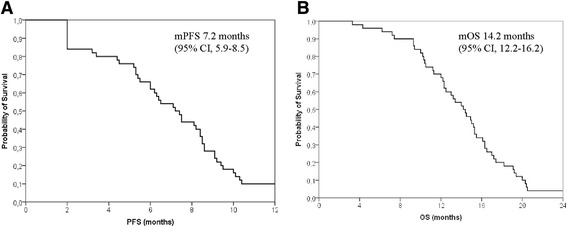
**Kaplan-Meier survival curves regarding A) PFS and B) OS.**

Median PFS (mPFS) was 7.2 months in the entire population (95% CI, 5.9-8.5) (Figure 1), 7.5 months in patients treated with anthracycline-containing regimens (95% CI, 5.5-9.5), and 6.5 months in patients treated with CMF (95% CI, 5.0-8.0). Five patients (10%) were free from disease progression after 1 year.

Median OS (mOS) was 14.2 months in the entire population (95% CI, 12.2-16.2) (Figure 1), 14.9 months in patients treated with anthracycline-containing regimens (95% CI, 12.8-17.0), and 13.0 months in patients treated with CMF (95% CI, 9.6-16.4). One-year survival rate was 68% in the entire population, 71.4% in patients treated with anthracycline-containing regimens, and 53.3% in patients treated with anthracycline-free regimens.

Irrespective of the clinical outcome analyzed, the observed differences between anthracycline-containing and anthracycline-free regimens were not statistically significant.

## Discussion

In this study, we reported on the efficacy of chemotherapy, consisting of three-drug anthracycline-containing and anthracycline-free regimens, in a series of 50 mMBC pretreated with endocrine treatments. To our knowledge, this is the largest series describing the efficacy of chemotherapy in this population.

In order to put our results into context, some intrinsic pitfalls firstly need to be discussed. The retrospective nature of our study ranks first. Unfortunately, lack of prospective data from randomized trials chronically plague the clinical management of these patients. To stress the concept that carrying out prospective studies in mMBC is extremely challenging, as already outlined elsewhere [2,22], poor accrual forced to prematurely close a small-sized study initiated by the SWOG cooperative group (SWOG-S0511, ClinicalTrials.gov; ID: NCT00217659). Recently, both the German Breast Group (ClinicalTrials.gov; ID: NCT01638247) and the European Organization for Research and Treatment of Cancer (ClinicalTrials.gov; ID: NCT01101425) promoted research in MBC. However, while attention is focused on hormonal therapy in the first case, the latter, to our knowledge, does not envision prospective, interventional trials but rather predominantly focuses on clinical and molecular characterization. We and others have recently discussed some strategies for overcoming this hurdle, such as including a pre-specified number of mMBC patients into prospective FBC trials [2,22]. In our opinion, however, this approach best fits with “small and smart” studies aimed to identify “exceptional” responders in a background of oncogene addiction, rather than with chemotherapy-focusing investigations. More realistically, we would like to encourage clinicians to collect information on the use of chemotherapy in the metastatic setting in order to strength our data and promote pooled analyses.

The heterogeneity in the modalities used for assessing disease extension and evolution, encompassing both imaging techniques and response criteria [20,21], deserves to be mentioned. We are aware that the efficacy of chemotherapy was not exactly captured in our study. Nonetheless, with an ORR of 56% encouraging signs of antitumor activity were registered.

Finally, the impossibility to retrieve safety data should be considered, with the solely exception of a fraction of patients treated in the most recent years, owing to the wide time window considered. Based on currently available, albeit incomplete, data we did not observe any unexpected warnings in terms of toxicity and adherence to therapy.

Looking at the data herein presented from a different angle the message conveyed is that established chemotherapy regimens commonly used in the female setting are also effective in mMBC patients after endocrine therapies. Thus, for patients with good performance status a series of conditions legitimize, in our opinion, the delivery of palliative chemotherapy including progression after multiple endocrine treatments, unacceptable hormone therapy-related side effects, rapidly progressive lesions, or lack of hormone receptor expression.

## Conclusions

Chemotherapy with anthracycline-containing and anthracycline-free regimens appears an effective treatment option for mMBC patients previously treated with endocrine therapy.

## Abbreviations

BC: Breast cancer
CMF/iv: Intravenous cyclophosphamide, methotrexate and 5-fluorouracil
CMF/oral: Oral cyclophosphamide, methotrexate and 5-fluorouracil
CR: Complete response
DCR: Disease control rate
FAC: 5-fluorouracil, doxorubicin and cyclophosphamide
FBC: Female breast cancer
FEC: 5-fluorouracil, epirubicin and cyclophosphamide
MBC: Male breast cancer
mMBC: Metastatic male breast cancer
mOS: Median overall survival
mPFS: Median PFS
ORR: Overall response rate
OS: Overall survival
PD: Progressive disease
PFS: Progression-free survival
PR: Partial responses
SD: Stable disease
TEC: Docetaxel, epirubicin and cyclophosphamide
